# Bacterial co-expression of human Tau protein with protein kinase A and 14-3-3 for studies of 14-3-3/phospho-Tau interaction

**DOI:** 10.1371/journal.pone.0178933

**Published:** 2017-06-02

**Authors:** Kristina V. Tugaeva, Philipp O. Tsvetkov, Nikolai N. Sluchanko

**Affiliations:** 1 A.N. Bach Institute of Biochemistry, Federal Research Center “Fundamentals of Biotechnology” of the Russian Academy of Sciences, Moscow, Russian Federation; 2 Department of biochemistry, School of Biology, Moscow State University, Russian Federation; 3 Aix-Marseille University, Inserm, CRO2 UMR_S 911, Faculté de Pharmacie, Marseille, France; 4 Institute of General Pathology and Pathophysiology, RAMS, Moscow, Russian Federation; 5 Department of Biophysics, School of Biology, Moscow State University, Moscow, Russian Federation; Biomedical Sciences Research Center Alexander Fleming, GREECE

## Abstract

Abundant regulatory 14-3-3 proteins have an extremely wide interactome and coordinate multiple cellular events via interaction with specifically phosphorylated partner proteins. Notwithstanding the key role of 14-3-3/phosphotarget interactions in many physiological and pathological processes, they are dramatically underexplored. Here, we focused on the 14-3-3 interaction with human Tau protein associated with the development of several neurodegenerative disorders, including Alzheimer’s and Parkinson’s diseases. Among many known phosphorylation sites within Tau, protein kinase A (PKA) phosphorylates several key residues of Tau and induces its tight interaction with 14-3-3 proteins. However, the stoichiometry and mechanism of 14-3-3 interaction with phosphorylated Tau (pTau) are not clearly elucidated. In this work, we describe a simple bacterial co-expression system aimed to facilitate biochemical and structural studies on the 14-3-3/pTau interaction. We show that dual co-expression of human fetal Tau with PKA in *Escherichia coli* results in multisite Tau phosphorylation including also naturally occurring sites which were not previously considered in the context of 14-3-3 binding. Tau protein co-expressed with PKA displays tight functional interaction with 14-3-3 isoforms of a different type. Upon triple co-expression with 14-3-3 and PKA, Tau protein could be co-purified with 14-3-3 and demonstrates complex which is similar to that formed *in vitro* between individual 14-3-3 and pTau obtained from dual co-expression. Although used in this study for the specific case of the previously known 14-3-3/pTau interaction, our co-expression system may be useful to study of other selected 14-3-3/phosphotarget interactions and for validations of 14-3-3 complexes identified by other methods.

## Introduction

Protein-protein interactions play a key role in a major number of biological processes by mediating multiple cellular functions and mechanisms. An important class of protein-protein interactions relies on specific recognition of post-translational modifications of proteins by special regulatory proteins. 14-3-3 is a family of relatively small (~30 kDa) eukaryotic regulatory proteins recognizing specific patterns that include phosphoserine or phosphothreonine [1,2]. By this interaction 14-3-3 proteins regulate structure, stability, intracellular localization, and interaction of their targets with other factors [3]. In different species, the 14-3-3 family is usually represented by several similar isoforms named with Greek letters and encoded by separate genes. In humans, there are seven isoforms (β, γ, σ, ζ, η, ε, τ/θ) that can exchange subunits and form homo- and hetero-dimers [4,5]. Most of the regulatory functions of 14-3-3 proteins are conducted *via* its dimeric form [3,4]. Still, in response to phosphorylation at dimerization interface [6–8], 14-3-3 dimers can dissociate and produce monomers with distinct properties [9] and poorly understood roles [4,10]. The dimers of 14-3-3 are better studied. They are involved in regulation of apoptosis, cell proliferation, signal transduction, ion channels trafficking, etc. [3], highlighting the importance of 14-3-3/phosphotarget interaction in many physiological and pathological processes.

14-3-3 proteins are abundant and have an extremely wide interactome, accounting for several hundreds of interaction partners [11,12]. Although the binding principle for 14-3-3/phosphotarget interactions seems to be universal [1,2], 14-3-3 proteins display selectivity and simultaneously coordinate multiple cellular processes [11,13]. Notwithstanding the key role of 14-3-3/phosphotarget interactions in number of vital cell processes, they are dramatically underexplored. Indeed, despite extensive studies of 14-3-3 interaction with phosphotargets, only few atomic structures of complexes have been solved [14,15]. Currently, the progress in the field is mostly challenged by the vast presence of intrinsically disordered regions in 14-3-3 partners [16], decreasing chances of successful crystallization and structural analysis [15].

Here, we focused on the 14-3-3 interaction with human Tau protein associated with the development of several neurodegenerative disorders, including Alzheimer’s and Parkinson’s diseases [17,18]. Microtubule-associated protein Tau is an intrinsically disordered protein [19,20], which binds tubulin [21] and regulates microtubule stability, axonal transport, and neurite outgrowth [22]. Tau/Tubulin interaction in turn is strictly regulated by Tau phosphorylation [22,23]. Being hyperphosphorylated in some diseases, known as tauopathies, Tau aggregate into filaments and form intracellular inclusions [22,24–26]. There are multiple protein kinases capable of Tau phosphorylation and tens of potential phosphorylation sites in this protein, making the overall picture of its regulation by phosphorylation extremely complicated [27]. Recently, it was shown that protein kinase A (PKA)-dependent Tau phosphorylation triggers its tight interaction with the full-length 14-3-3ζ [28,29] and that there are several phosphorylation sites which contribute to 14-3-3 binding [18,30]. Lately, structures of phosphopeptide complexes with two of these sites of Tau and the C-terminally truncated 14-3-3σ have been reported [18]. However, the stoichiometry and mechanism of the 14-3-3 interaction with phosphorylated Tau (pTau) are not clearly elucidated.

In this work, we describe a simple co-expression system aimed to facilitate the biochemical and structural studies on the 14-3-3/pTau interaction. We show that co-expression of Tau with PKA results in phosphorylation of the established earlier and also novel potential 14-3-3 binding sites which were not previously considered in this context but were reported as phosphorylatable *in vitro* and *in vivo*. Co-expressed and phosphorylated Tau displays similar ability to interact with different 14-3-3 isoforms as the protein phosphorylated *in vitro*. The described co-expression system may be also useful for studies of other selected 14-3-3/phosphotarget interactions.

## Materials and methods

### Chemicals

All chemicals were of the highest quality and purity available. Milli-Q quality (18.2 MΩ/cm) water was used for preparation of all solutions which were filtered (0.2 μm) before use.

### Cloning of components for bacterial co-expression

The cDNA of 14-3-3σ (Uniprot ID P31947; residues 1–231 out of 248) was subcloned into the second multiple cloning site (MCS2) of the CDFduet-1 vector by using *Pfu* DNA-polymerase, *NdeI/XhoI* sites and the following forward (5’-ATATACATATGGAGAGAGCCAGTCTG-3’) and reverse (5’-ATATACTCGAGTCACGTCCACAGTGTCAGG-3’) primers (sites for restriction endonucleases are underlined); the cDNA of the catalytic subunit of mouse PKA was moved from pET15 vector [31] into pACYCduet-1 vector using *NcoI/BamHI* sites (MCS1); the cDNA of human fetal Tau isoform containing three repeats (3R-Tau) in the microtubule binding domain (Uniprot ID P10636-2; 352 residues) was moved from pET23 plasmid [29] into pET28-His-3C vector [15] using *NdeI/XhoI* sites (Table 1) to get the in-frame inserts without using PCR (Table 1). As a result, Tau construction had the N-terminal MEHHHHHHLEVLFQ↓GPH sequence with the His_6_-tag and 3C cleavage site (underlined). The integrity and correctness of the constructions obtained was verified by DNA sequencing (http://evrogen.com/). The plasmids obtained in this study are available upon request. The intact pACYCduet-1 and CDFduet-1 vectors were kindly provided by York structural biology laboratory (York, UK).

**Table 1:** Components of the co-expression system.

Component	Description	Vector name	Origin of replication	Antibiotic resistance	Tag	Cleavable by	Calculated mass, kDa
14-3-3	Human 14-3-3σ lacking C-terminal flexible peptide (residues 232–248)	CDFduet-1 MCS2 (*NdeI/XhoI*)	CDF	Streptomycin	no	–	26.1
Protein kinase	Catalytically active subunit of mouse PKA	pACYCduet-1 MCS1 (*NcoI/BamHI*)	P15A	Chloramphenicol	His_6_	Thrombin	44.9
Tau protein	Fetal isoform of human Tau protein	pET28-His-3C (*NdeI/XhoI*)	pBR322	Kanamycin	His_6_	3C*	38.9 (36.8)**

*Note, that His_6_-MBP-3C protease (~69.7 kDa), suitable for the subtractive IMAC [33], was used (see details in the text).

**- without the His-tag. Note, that the electrophoretic mobility of Tau corresponds to 48–52 kDa depending on its phosphorylation status.

### Protein expression and purification

To transform “chemically” competent *Escherichia coli* BL21(DE3) cells, which had no background resistance to chosen antibiotics, the obtained plasmids (Table 1) were used for co-transform either simultaneously or consecutively. Both transformation schemes resulted in similar levels of protein expression. However, in case of consecutive co-transform it is necessary to make the cells competent after each round of transformation. This makes procedure more flexible, and further transformation becomes more efficient.

Single colonies of transformed cells were used to produce “chemically” competent cells or to start test expressions. In the latter case, 0.3 ml out of 20 ml of an overnight culture in Luria-Bertani (LB) media (Amresco) were inoculated into 20 ml of fresh LB media with appropriate antibiotics. Culture grown until OD_600_ reached 0.6–0.7 and then isopropyl-β-thiogalactoside (IPTG) (Helicon) was added to a final concentration of 1 mM for 4 h or overnight cultivation at 37°C. The most successful clones were stocked at -80°C in 20% glycerol and used for preparative expression in 3 L flasks containing 1 L of LB media with appropriate antibiotics under conditions optimized during test expressions.

To harvest cells, bacterial culture was centrifuged (1h, 3250g), then the pellet was suspended in 30–40 ml of an ice-cold buffer A (30 mM Tris-HCl, pH 8.0, containing 120 mM NaCl, 10 mM imidazole, 0.05 mM phenylmethanesulfonyl fluoride (PMSF), 0.001 mM leupeptin, 0.001 mM pepstatin) and stored at -80°C or immediately used for protein purification. We used near-physiological 120 mM salt concentration to maximize chances of preserving 14-3-3/phosphotarget complexes formed in cells upon co-expression. Cell suspensions were sonicated on ice, centrifuged at 4°C for 1 h at 12,000g and filtered through the 0.22 μm Millipore membrane to clarify supernatant with the overexpressed proteins. Importantly, all components of the co-expression system were completely soluble.

**Fig 1:**
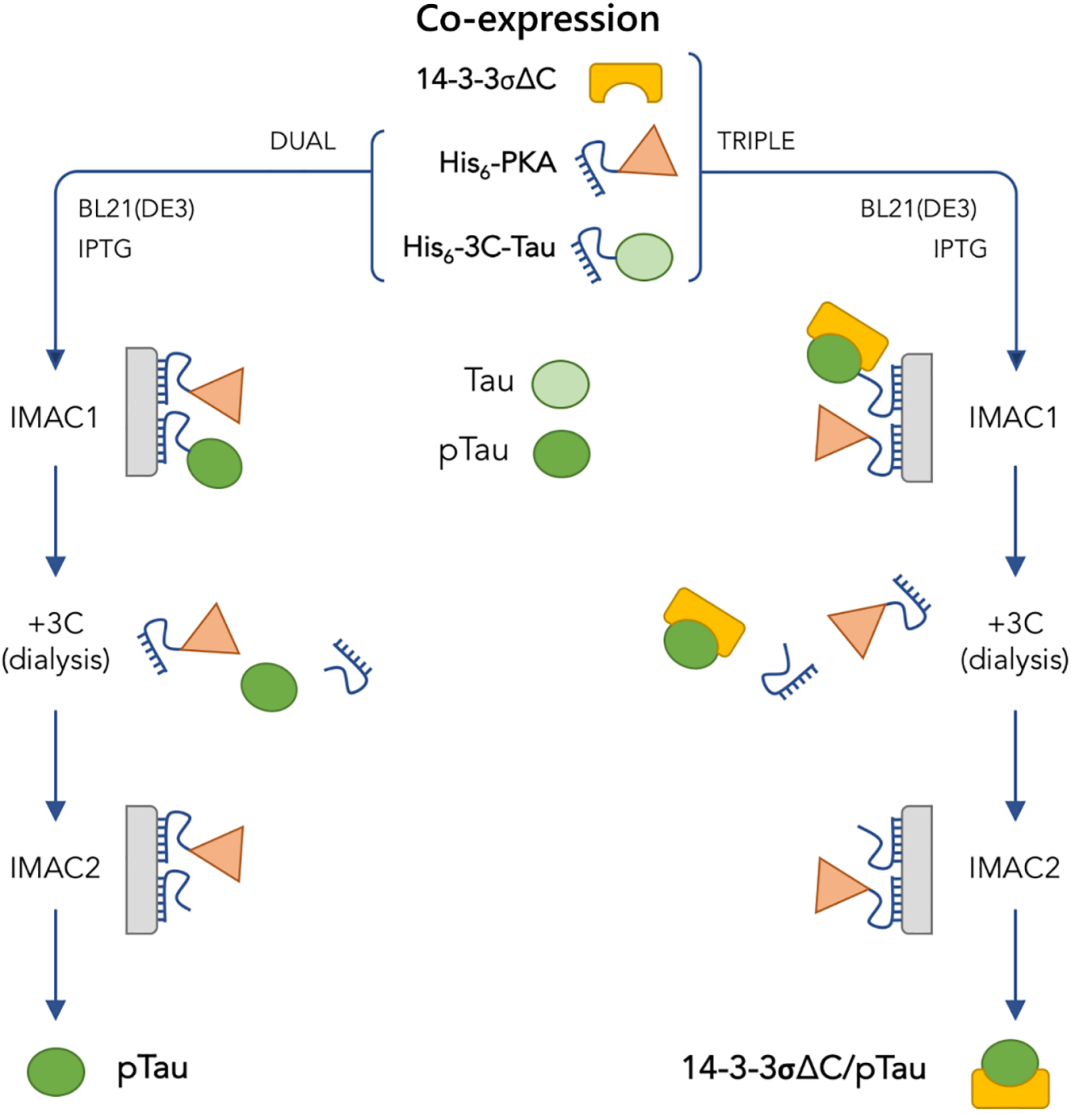
Co-expression system proposed. Scheme demonstrates the pipelines in the case of the proposed dual (left side) or triple (right side) co-expression/purification system dealing with production of a phosphotarget or a 14-3-3/phosphotarget complex, respectively, using Tau protein as an example. Protein components of the system are coded by yellow rectangle (14-3-3), orange triangle (PKA), and green oval (Tau); light and dark oval stand for unphosphorylated and phosphorylated Tau, respectively. The His-tag on PKA and Tau is shown by a short blue curves. Note, that 3C protease was removed during IMAC2 because it contained an uncleavable His_6_-tag. IMAC stands for immobilized metal-affinity chromatography.

The supernatant was loaded on a HisTrap column (GE Healthcare) equilibrated with buffer A and, after washing with 10 mM imidazole, bound proteins were eluted with buffer A additionally containing 500 mM imidazole (dual co-expression) or with a 10–500 mM imidazole gradient (triple co-expression) on buffer A (see Fig 1). In the cases of triple co-expression, we used low flow rates (0.5 ml/min) at the loading and washing steps to minimize shear forces potentially damaging to protein complexes with untagged 14-3-3. In the case of dual co-expression, these precautionary measures are less important. Proteins bound to the HisTrap column on the first immobilized metal affinity chromatography (IMAC) (Fig 1) were treated with 3C protease (substrate/protease ratio of 100/1 assessed by optical density at 280 nm) to cleave off the His-tag on Tau protein directly in a dialysis tube, while being dialyzed overnight at 4°C against 2 L of 20 mM Tris-HCl buffer (pH 7.5) containing 120 mM NaCl, 0.1 mM ethylenediaminetetraacetic acid (EDTA), 0.1 mM PMSF, 2.5 mM β-mercaptoethanol (ME). Dialysate was clarified by centrifugation for 30 min, 12,000g at 4°C and then subjected to the second IMAC to separate highly pure pTau or 14-3-3/pTau complexes from the mixture of His_6_-tagged 3C protease, PKA, and protein contaminants from the previous IMAC step (see Fig 1). Protein composition in different fractions was analyzed using sodium dodecyl sulfate polyacrylamide gel-electrophoresis (SDS-PAGE) [32]. If necessary, protein samples were concentrated on Amicon concentrators (Millipore) with 3 kDa cut-off.

In the case of Tau protein, which is a natively unfolded and heat-stable protein, as an alternative means of purification, we could use a heat treatment (95°C; 25 min) followed by subtractive IMAC, however, this procedure most likely is not suitable for other 14-3-3 targets.

In order to identify sites of Tau protein phosphorylated upon bacterial co-expression with PKA, we performed in-gel trypsinolysis and further analysis of the obtained tryptic fragments by mass spectrometry on a matrix-assisted laser desorption ionization time-of-flight/time-of-flight (MALDI TOF/TOF) ultrafleXtreme mass-spectrometer (Bruker, Germany) essentially as reported earlier [8,15,33]. Briefly, the band was excised out of the gel and immersed in the MS-grade trypsin solution for 1 or 24 h, and, after desalting, MS spectra were recorded in the reflectron mode to reveal potential phosphopeptide masses using the Mascot search. The highest scoring masses corresponding to predicted tentative phosphopeptides (with peaks over 4000 a.u. for 1–2 kDa or over 2000 a.u. for >2 kDa) were then confirmed in the tandem regime. The spectra were assigned and analyzed in flexAnalysis 3.3 software (Bruker, Germany).

The full-length human 14-3-3γ (Uniprot ID P61981) and 14-3-3ζ (Uniprot ID P63104) were expressed and purified as described earlier [34], [35]. The monomeric 14-3-3ζ mutant (m58E) was obtained in the previous work [8]. The untagged human 14-3-3σ (Uniprot ID P31947), devoid of the C-terminal flexible peptide (residues 232–248), was purified according to the procedure described for 14-3-3ζ [35] using ammonium sulfate fractionation and a combination of anion-exchange and gel-filtration chromatography. The His_6_-tagged human 14-3-3ε (Uniprot ID P62258) with a tobacco etch virus (TEV)-cleavable His-tag was obtained by subtractive immobilized metal-affinity chromatography (IMAC) and gel-filtration. The human rhinovirus 3C protease was obtained as the His_6_-MBP-fusion according to the scheme described in previous work [33]. For *in vitro* phosphorylation assays, PKA was obtained in one step using IMAC [36]. Thermosensitive alkaline phosphatase (FastAP) was obtained from ThermoFisher Scientific. All individually purified proteins were homogeneous according to the SDS gel-electrophoresis [32].

Protein concentration was determined spectrophotometrically on a NanoPhotometer P330 (Implen) using calculated in ProtParam (http://www.uniprot.org/) molar extinction coefficients at 280 nm equal to 7450 M^-1^ cm^-1^ for Tau, 31860 M^-1^ cm^-1^ for 14-3-3γ, 27390 M^-1^ cm^-1^ for 14-3-3ζ, 28880 M^-1^ cm^-1^ for 14-3-3ε, 25900 M^-1^ cm^-1^ for 14-3-3σ∆C.

### Protein phosphorylation and dephosphorylation *in vitro*

To reveal any undesired consequences of phosphorylation, four purified isoforms of human 14-3-3 (14 μM per monomer) were incubated with large amounts of PKA (2 μM) in buffer P (50 mM Hepes-NaOH, pH 7.3, containing 10 mM NaCl, 4 mM MgCl_2_, 60 μM ATP) in a total volume of 110 μl for 2 h at 30°C and then analyzed by gel-electrophoresis under non-denaturing conditions at pH 8.6 [37]. Dephosphorylation of purified pTau^coex^ (0.2 mg/ml) was performed by thermosensitive alkaline phosphatase FastAP (ThermoFisher Scientific) in a buffer recommended by manufacturer, but devoid of bovine serum albumin (10 mM Tris-HCl, pH 8.0, 5 mM MgCl_2_, 100 mM KCl), for 1.5 h at 37°C and then analyzed by SDS gel-electrophoresis [32].

### Analysis of the 14-3-3/pTau interaction

To test the ability of pTau^coex^ to interact with 14-3-3, we first mixed purified Tau from dual co-expression with PKA with either 14-3-3 isoform (1.25 mg/ml) in buffer A for 30 min at 30°C and then analyzed by gel-electrophoresis (350 V; 50 min) under non-denaturing conditions at pH 8.6 in 15% polyacrylamide gels [37]. For comparison, 14-3-3/pTau complex or pTau obtained by triple or dual co-expression with PKA were also analyzed. To ensure that the observed protein band corresponded to the 14-3-3/pTau complex, we excised it from the native gel, mechanically ground it, resuspended in the SDS-containing buffer, and then analyzed by SDS gel-electrophoresis versus 14-3-3 and pTau controls.

Alternatively, 14-3-3ζ (10 μM), pTau^coex^ (15 μM), or their mixture at indicated protein concentrations were pre-incubated for 20 min at 37°C and then subjected to size-exclusion chromatography on a Superdex200 Increase 10/300 column (GE Healthcare) operated at room temperature and a flow rate of 1.2 ml/min. The profiles were followed by absorbance at 227 nm since at 280 nm the extinction coefficient of Tau is aproximetly 5 times lower than that of 14-3-3.

## Results and discussion

### Design of elements for Tau protein co-expression with PKA and 14-3-3

In order to create a flexible and convenient co-expression system for studies on 14-3-3/phospho-Tau interaction and probably other targets of 14-3-3, all three components (protein kinase, 14-3-3, and target) had to be carefully designed and cloned in different vectors having compatible origins of replication and antibiotic resistance.

#### Choice of protein kinase

Often protein kinase A (PKA) works in pair with 14-3-3 since it phosphorylates target proteins at (R/K)RX(S/T) sequences (where X is any amino acid) [38], which are also a part of the consensus motifs (R/K)X_2-3_(pS/pT)X(P/G) for 14-3-3 binding [2]. Due to this overlap, PKA phosphorylation often *prepares* targets for 14-3-3 binding. Moreover, the well-established expression of the soluble catalytically active subunit of PKA in *Escherichia coli* [31,36], makes this kinase a robust phosphorylating instrument for protein engineering. Recently, a 14-3-3 phosphotarget, the small heat shock protein HSPB6 (HSP20), has been successfully produced in bacterial co-expression with protein kinase A [15] or protein kinase G [39]. Importantly, although being able to phosphorylate Ser16 and Ser59 of HSPB6 *in vitro* [40,41], PKA bacterially co-expressed with HSPB6 resulted in a single phosphorylation of only Ser16 [15], suggesting that conditions for phosphorylation *in vitro* and in cells can be different, probably, because of the crowding effects of cellular environment. To our knowledge, triple co-expression of a target, PKA, and 14-3-3 has not been tested yet.

#### Tau protein construct

For our co-expression system, we considered an established 14-3-3 partner that could be readily phosphorylated by PKA and produced in *E*. *coli* in the soluble form. Tau protein meets all these criteria as it is (i) a “good” substrate for PKA [42], (ii) a well-known 14-3-3 partner [18,28–30,43], and is also (iii) soluble upon bacterial expression. The fetal isoform of human Tau (352 residues) containing several potential 14-3-3 binding sites was used.

#### Design of 14-3-3 construct

Not only 14-3-3 targets but some 14-3-3 isoforms itself can be phosphorylated by PKA. This mainly involves a semiconservative Ser58 (RXXS^58^W, ζ isoform numbering) located in the dimerization interface of 14-3-3 and, expectedly favor dimer dissociation [6,8,35,44]. Although phosphorylation level of 14-3-3 dimers is quite low [8,34,45], utilization of 14-3-3 isoforms and the choice of the appropriate 14-3-3 variant for a co-expression system with PKA required careful consideration. Fortunately, some of the 14-3-3 isoforms, including 14-3-3τ/θ and 14-3-3σ, contain natural S→A substitution at position 58 and therefore are favorable candidates. Despite 14-3-3τ/θ is more abundant in the brain, 14-3-3 isoforms have significantly overlapped interactome, and another advantage of 14-3-3σ, besides the absence of Ser58, was also substantiated by the need to use the co-expression system for structural biology studies, where 14-3-3 complexes involving 14-3-3σ crystallized most readily at least in several instances (unpublished observations and [15]). Currently, the role of the flexible C-terminus of 14-3-3 is not fully clear. Since its absence in the 14-3-3 constructs does not prevent binding of genuine phosphopartners [15,18,46] but its presence may interfere with protein crystallization and may play some autoinhibitory role [47,48], for our co-expression studies we decided to use the engineered σ isoform lacking the C-terminus (14-3-3σΔC). This construct has been successfully used in a range of structural studies as a binding competent [15,18,46].

**Fig 2:**
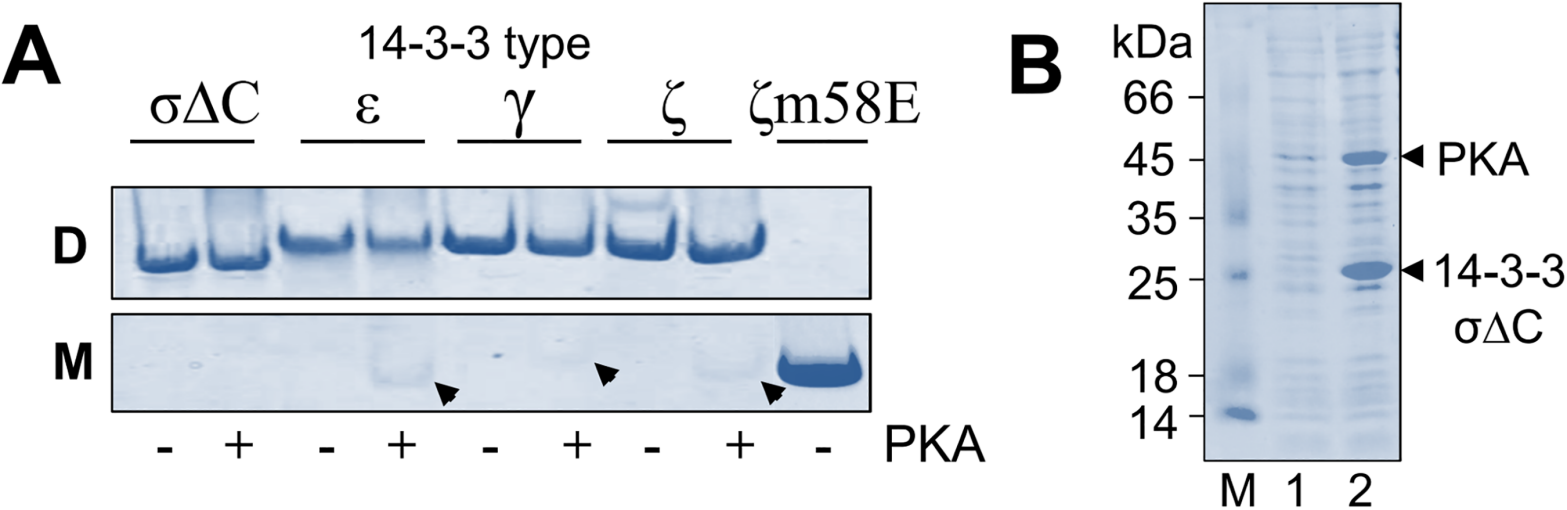
Selection of 14-3-3 variant for co-expression with PKA. **A.** Analysis of the results phosphorylation of various 14-3-3 variants in the absence (“-”lanes) or in the presence (“+” lanes) of PKA by native gel-electrophoresis in a 15% polyacrylamide gel shown separately for gel regions containing dimeric (“D”) and monomeric (“M”) protein forms. The monomers formed in the case of ε, γ, and ζ are marked by arrows. Monomeric 14-3-3ζ mutant m58E was used as a control. See Materials and methods for details. **B.** Test co-expression of 14-3-3σΔC and PKA in BL21(DE3) cells. Lane M represents molecular mass standards. The samples before (lane 1) and after (lane 2) IPTG induction were analyzed by SDS gel-electrophoresis. The gels were stained by Coomassie brilliant blue.

We performed *in vitro* phosphorylation of four 14-3-3 isoforms (ε, γ, ζ, and σΔC) at a large PKA amount (See Materials and methods) to exaggerate probable consequences of their phosphorylation upon co-expression with PKA, and analyzed the results using a native gel-electrophoresis under established conditions [8]. We found that ε, γ, and ζ isoforms displayed a different extent of phosphorylation-induced protein monomerization (Fig 2A), which is in line with the presence of phosphorylatable Ser58 in dimerization interface of all these isoforms. Although the level of monomers was not very high, it may not be optimal to use any of these variants for co-expression with PKA because undesired phosphorylation-induced monomerization can still take place, directly and indirectly affecting phosphopartner binding. Under the same conditions, 14-3-3σΔC was resistant to phosphorylation-induced monomerization (Fig 2A), indicating that this variant is more suitable for co-expression with PKA. Indeed, co-transformation and co-expression of CDF-14-3-3σΔC and pACYC-PKA revealed IPTG-induced expression of major ~26 kDa and ~45 kDa bands corresponding to these proteins (Fig 2B), however, we could not detect any significant 14-3-3σΔC phosphorylation (data not shown). Thus, we have created PKA/14-3-3σΔC expressing cells competent and can use them for transformation with a compatible plasmid of the 14-3-3 target under study.

#### Choice of vectors

We chose vectors with compatible origins of replication that allow independent selection on differing antibiotics (Table 1). Although developed here for 14-3-3/pTau interaction, the proposed system is very flexible and in principle permits combining almost any desired 14-3-3 phosphotarget with PKA/14-3-3 cells, provided that a vector for a phosphotarget is compatible with those of 14-3-3 and PKA (see Table 1). For example, most common pBR322 vectors conferring resistance to kanamycin or ampicillin are compatible. Preferably, the electrophoretic mobility of a potential 14-3-3 partner should be different from 26 or 45 kDa to facilitate its identification in cell lysate as a band distinct from that of 14-3-3 or PKA. In the simplest case, it is recommended to sub-clone a phosphotarget of interest into the same pET28-His-3C vector as used for Tau protein in this study.

For efficient purification and separation of 14-3-3/phosphotarget complexes, we used cleavable hexahistidine tags in the N-termini of PKA and the 14-3-3 target, differing by cleaving enzymes (Table 1). Importantly, 14-3-3 did not contain a His-tag and therefore did not bind to a HisTrap column on its own (data not shown). All the components had different theoretical masses making them readily identified on the SDS gel-electrophoresis upon simultaneous expression. For a general use, the 3C cleavable His-tag should be considered attached to a least abundant component (either 14-3-3 or its target) to ensure saturation of a His-tag containing component by excessive amounts of its untagged partner and more efficient separation of the complex on IMAC1.

### Dual co-expression of Tau protein and PKA

#### Phosphorylation of human Tau upon co-expression with PKA

**Fig 3:**
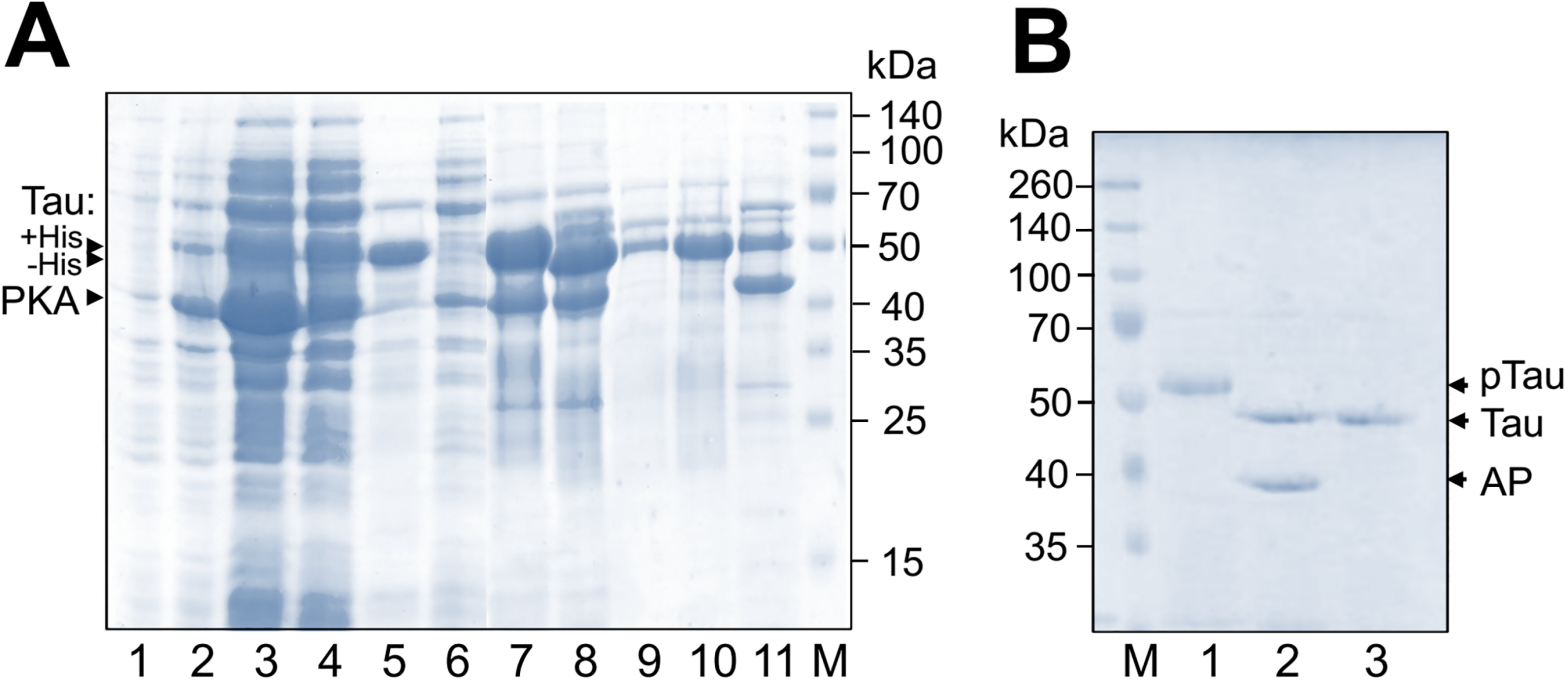
Obtaining of ready-to-use phosphorylated Tau protein by its co-expression with PKA. **A.** Step-by-step procedure of protein expression and purification. Uninduced (lane 1), or IPTG-induced sample (lane 2), total (lane 3) and soluble (lane 4) fractions, heat-treated (15 min at 90°C) and centrifuged lysate (lane 5), IMAC1 flowthrough (lane 6) and bound (lane 7) fractions, 3C-cleaved fraction (lane 8; note the downward shift of the Tau band) loaded on IMAC2 and corresponding IMAC2 wash at 10 mM (lane 9) or 20 mM imidazole (lane 10) or 500 mM imidazole elution (lane 11) fractions are shown. M, mass standards (shown in kDa to the right). **B.** Tau co-expressed with PKA was purified (lane 1) and dephosphorylated (lane 2) by FastAP phosphatase (AP). Dephosphorylated Tau was separated from AP by heat treatment for 15 min at 90°C (lane 3). M–molecular mass standards (indicated in kDa). Assigned positions of proteins are shown on panels A, B by arrows. The gels were stained by Coomassie brilliant blue.

Co-transformation of competent BL21(DE3) cells by compatible plasmids encoding Tau and PKA or transformation of competent BL21(DE3) cells already carrying PKA plasmid (see Fig 1, left) resulted in visible induction of ~45 kDa and ~52 kDa protein bands upon addition of IPTG (Fig 3A). Since Tau is a naturally unfolded protein, purification schemes often include heat treatment [23]. In our case, Tau protein co-expressed with PKA remained in the supernatant even after 25 min boiling of the cell lysate, which helped to unequivocally assign its band (Fig 3A). The band of PKA (~45 kDa) disappeared after heat treatment due to its thermal lability. The band of Tau protein treated with phosphatase displayed a significant downward electrophoretic mobility shift (Fig 3B), a characteristic feature of Tau phosphorylated at C-terminal residues Ser320 and Ser327 [42]. This confirms that Tau protein was phosphorylated *in bacteria* by the co-expressed PKA subunit. Tau was cleaved with 3C protease to remove the His-tag that was detected by a shift on the SDS gel-electrophoresis (Fig 3A, lanes 7 and 8), and could be finally obtained as nearly homogeneous “flowthrough” fraction (at 20 mM imidazole) during the second IMAC (Fig 3). Interestingly, under conditions used and low concentrations of imidazole, Tau tended to bind to the Ni-Sepharose, most likely because of the unusually high level of positively charged residues in its sequence and 11 inherent histidines in particular.

**Fig 4:**
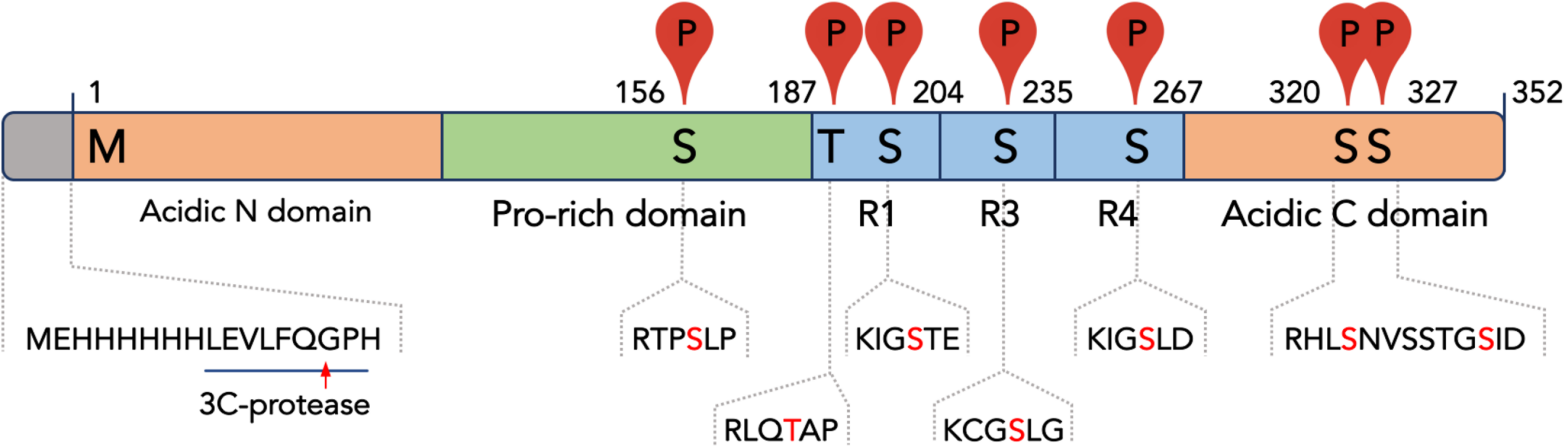
Fetal human Tau construction used in this study, with the most remarkable features indicated. Seven PKA phosphorylation sites confirmed in this study are shown by (P) with the surrounding residues shown below. The numbering corresponds to human fetal Tau. R1, R3, R4 denote tubulin-binding repeats. The names of the domains are shown below the sequence. The N-terminal His_6_-tag and the 3C cleavage site (underlined) are indicated.

Tau protein is highly phosphorylated in the brain of patients with different tauopathies, and phosphorylation patterns can be highly complicated [22,25,27]. Among different kinases able to phosphorylate Tau, PKA was reported to phosphorylate at least five residues in the fetal Tau isoform *in vitro*, namely Ser156, Ser235, Ser267, Ser320, Ser327 [42], however, phosphorylation level was found to be less than 2.5 [29,42]. It is important to note that Ser156, Ser235, and Ser267 were found to play a crucial role in Tau interaction with 14-3-3, and mutation at any of these sites significantly reduced the binding [30]. Taking this into account, we decided to carefully analyze phosphorylation status of Tau co-expressed with PKA by mass-spectrometry. We found that more than 90% of the His_6_-3C-Tau sequence can be covered and the N-terminus of the bacterially expressed Tau was intact. At the same time, in all the samples we failed to confirm the presence of last 3 amino acids QGL, most likely indicating intracellular proteolytic degradation of the expressed Tau. Still, the electrophoretic behavior suggested high purity and absence of contaminating proteolytic fragments usually present in all preparations of Tau obtained by classical methods [29]. Noteworthy, in terms of the absence of Tau fragments, our expression and purification protocol appears to be superior to that published very recently and utilizing truncated Tau mutants with a TEV-cleavable His-tag [49]. Mass-spectrometric analysis of the phosphopeptides revealed that pTau^coex^ is heavily phosphorylated involving at least 7 residues that we could validate by MS/MS (Table 2 and Fig 4). Phosphorylation of Ser320 and Ser327 could explain the electrophoretic shift [42] of the co-expressed Tau on the SDS gel (Fig 3B). We could also confirm phosphorylation of the primary 14-3-3-binding candidates, i.e. Ser156, Ser235, and Ser267 (Fig 4), we showed earlier [30]. Most importantly, we confirmed phosphorylation of Ser204 (site KIG**pS**TE), Thr187 (site RLQ**pT**AP), and Ser320 (site RHL**pS**NV) (Table 2 and Fig 4) that were reported to be phosphorylated by different protein kinases, not necessarily including PKA [23,50–55]. For instance, phosphorylation of Ser204, Ser235, Ser267, and Ser156 by PKA and MARK (microtubule affinity regulated kinase) was reported to significantly reduce the affinity of Tau to tubulin and inhibited Tau assembly into paired helical filaments (PHFs) [23]. Phosphorylation of Thr187 (site RLQ**pT**AP) was demonstrated by using Rho-dependent kinase (ROCK) [52], a crucial regulator of several important neuronal functions [56], and even by proline-directed mitogen-activated protein kinase p38 [53], however, could be phosphorylated to only low levels by PKA *in vitro* [23]. Intriguingly, this site overlaps with one of the stretches, LQTA, which is directly involved in Tau/tubulin binding (Fig 4) [57]. Moreover, despite direct binding of this phosphosite to 14-3-3, to our knowledge, has not been reported, its environment (RLQpTAP) ideally corresponds to the consensus 14-3-3-binding motifs (Table 2) [2], thus making it a novel promising candidate 14-3-3 site with strong physiological implications.

**Table 2:** Identification by in-gel trypsinolysis and tandem mass-spectrometry of phosphorylated sites within human fetal pTau^coex^ in *E*. *coli*. Numbering for two alternatively spliced Tau isoforms is shown. Likelihood that the identified phosphopeptide (underlined) serves as a 14-3-3-binding site is estimated from “-”(low probability) to “+++” (high probability), compared to the existing literature (right column). Sites of trypsinolysis is designated by “.”.

Sequenced Tau phosphopeptides	Mass, Da	Position in 352 a.a. (441 a.a.) human Tau isoform	Similarity with canonical 14-3-3 binding sites, R/KX_2-3_pS/pTXP/G	14-3-3 binding reported
R.SRTP**pS**^**156**^LPTPPTREPK.K	1743.9	Ser156 (Ser214)	+++	[18,28,30]
K.SRLQ**pT**^**187**^APVPMPDLK.N K.SRLQ**pT**^**187**^APVPMPDLKNVK.S	1632.8 1974.0	Thr187 (Thr245)	+++	n/a
K.IG**pS**^**204**^TENLKHQPGGGKVQIVYKPVDLSK.V	2972.6	Ser204 (Ser262)	+	n/a
K.CG**pS**^**235**^LGNIHHKPGGGQVEVK.S	1996.9	Ser235 (Ser324)	++	[18,28,30]
K.IG**pS**^**267**^LDNITHVPGGGNK.K	1658.8	Ser267 (Ser356)	++	[30]
R.HL**pS**^**320**^NVSSTG**pS**^**327**^IDMVDSPQLATLADEVSASLAK.Q	3403.3	Ser320 (Ser409)	++	n/a
		Ser327 (Ser416)	-	n/a

In summary, we established that pTau^coex^ is phosphorylated on not only several already known, but also on novel potential 14-3-3-binding sites that substantiated further utilization of pTau^coex^ for studies of its interaction with 14-3-3. At present, the hierarchy and interrelations between these multiple 14-3-3-binding sites are not well understood and lie before us to explore. The ability of a particular 14-3-3-binding site (e.g., Thr187) to be phosphorylated by several kinases may illustrate the necessity that its interaction with 14-3-3 is controlled by different intracellular stimuli, however, a particular enzyme phosphorylating it may not be crucial for structural studies of a corresponding 14-3-3/target complex as its phosphorylation *per se* in this case is more important.

#### Bacterially phosphorylated human Tau is competent for 14-3-3 binding

**Fig 5:**
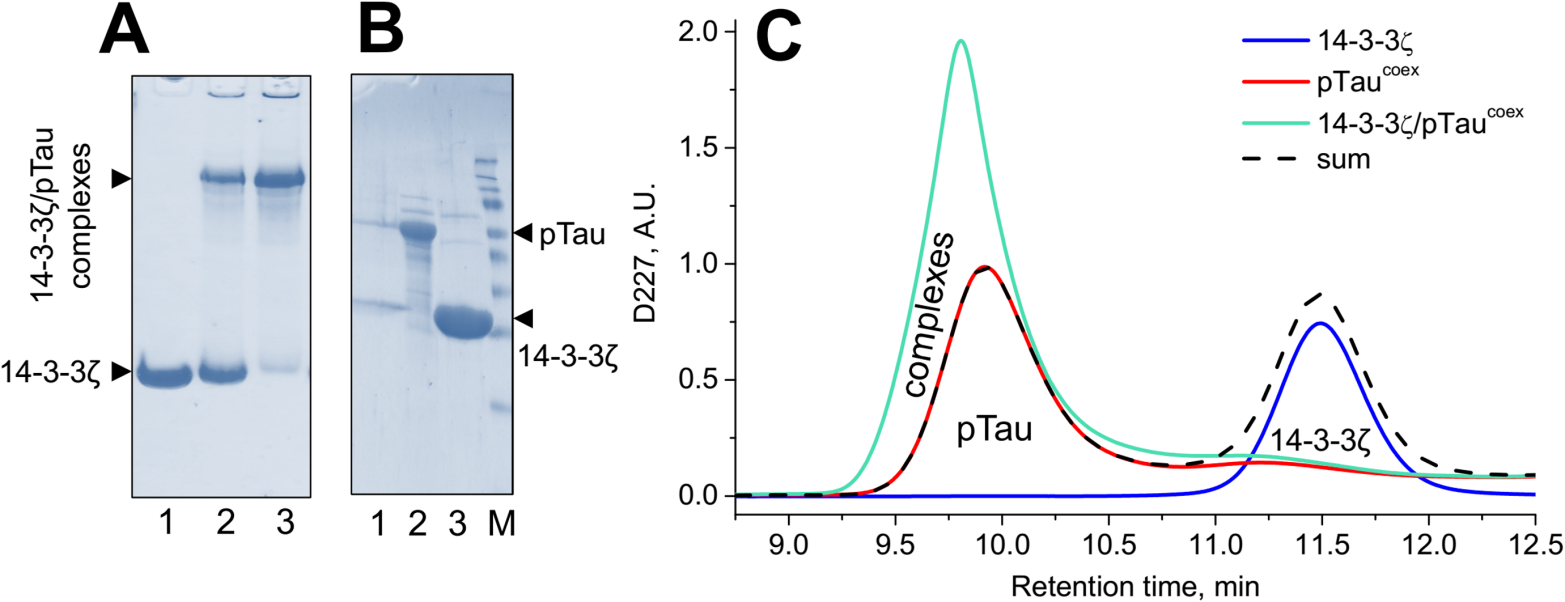
Validation of interaction between pTau^coex^ and 14-3-3ζ by means of native gel-electrophoresis and analytical size-exclusion chromatography. **A.** Purified 14-3-3ζ (lane 1) or its mixture with 1x (lane 2) or 2x (lane 3) amount of pTau^coex^ run on non-denaturing gel-electrophoresis in a 15% gel. 14-3-3ζ was present in equal amounts on lanes 1–3 but migrated to the intermediate (tentative complex) band in the presence of pTau which has pI>9 and does not enter the gel at pH 8.6. **B.** The tentative complex band was excised from the native gel and analyzed by SDS-PAGE to show the presence of both partners (lane 1). Lane 2, pTau^coex^ control; lane 3, 14-3-3ζ control; M, molecular mass standards (15, 25, 35, 40, 50, 70, 100, 140, 260 kDa). Arrows indicate positions of 14-3-3 and pTau. The gels were stained with freshly prepared Coomassie brilliant blue. **C.** Elution profiles of purified 14-3-3ζ, pTau^coex^, their mixture, or algebraic sum of the individual profiles followed by absorbance at 227 nm using a Superdex200 Increase 10/300 column (GE Healthcare) operated at a flow rate of 1.2 ml/min. At least three experiments were performed with essentially similar results.

On the next step, functionality of pTau^coex^ was confirmed by its ability to interact with 14-3-3. Purified pTau^coex^ was mixed with 14-3-3ζ at two ratios and then analyzed by native gel-electrophoresis (Fig 5A). This electrophoretic system is convenient for such an analysis [29,30] as it readily separates Tau possessing alkaline pI and low electrophoretic mobility, 14-3-3 possessing acidic pI and high mobility, and their complexes of intermediate mobility [29]. Supported by our previous observations [29,30] and by the presence of both proteins confirmed via the SDS gel-electrophoretic analysis of the excised protein band corresponding to the tentative complex (Fig 5B), this experiment clearly showed the ability of pTau preparation to tightly interact with 14-3-3ζ (Fig 5A). This result was further confirmed by analytical size-exclusion chromatography. While individual 14-3-3ζ and pTau^coex^ gave well-separated peaks at 11.49 and 9.93 min, respectively, the elution profile of their mixture was distinctly different from the sum of their individual profiles and was presented by a single peak with a maximum at 9.80 min. This peak had substantially larger amplitude than that of Tau and was shifted toward smaller retention times (Fig 5C), indicating the formation of the 14-3-3ζ/pTau complex.

**Fig 6:**
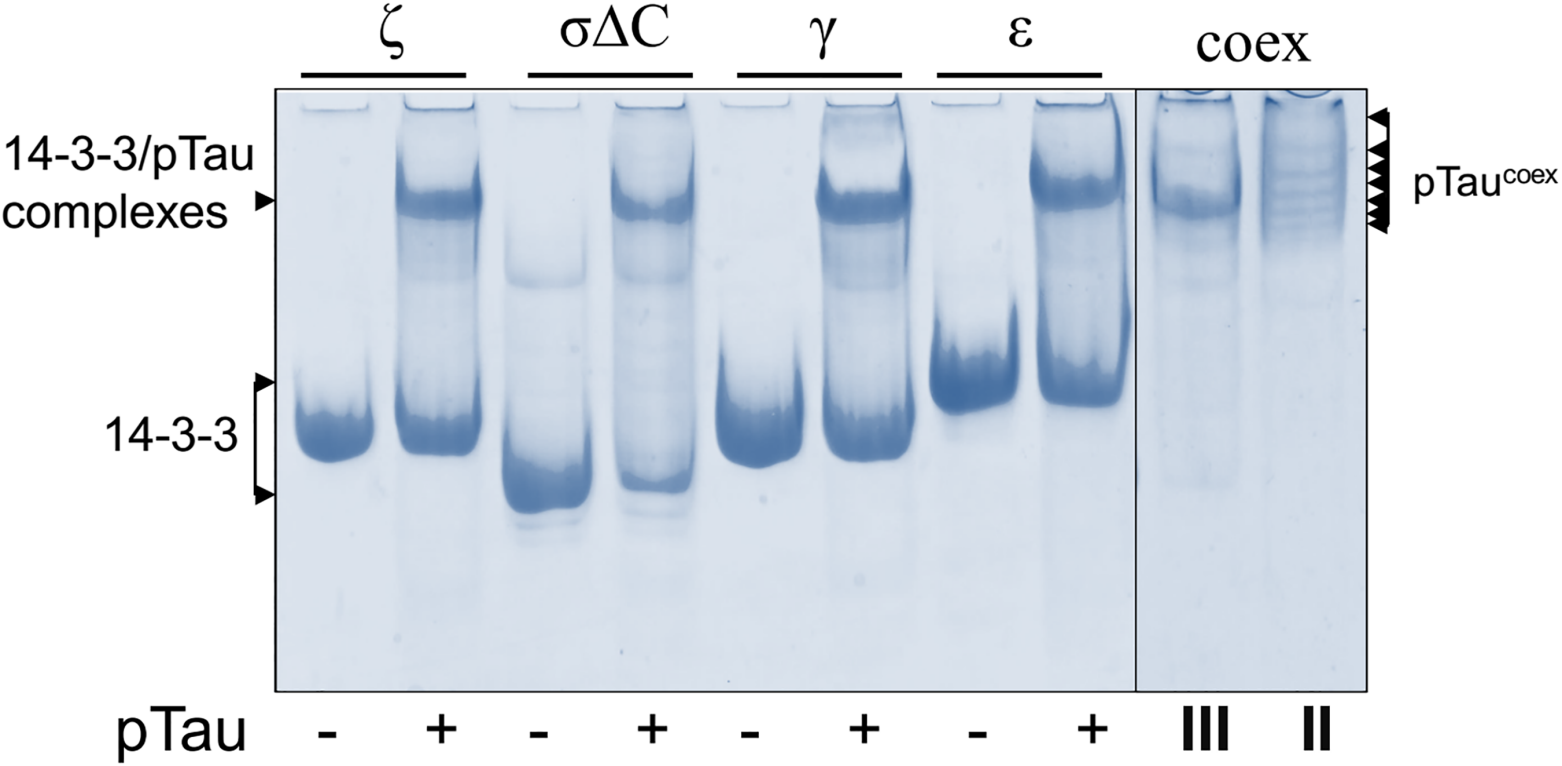
Interaction of different 14-3-3 isoforms and pTau^coex^ analyzed by means of native gel-electrophoresis. Left, odd lanes correspond to 14-3-3 isoforms alone and even lanes correspond to their mixtures with pTau^coex^. The upper bands contain both proteins (see Fig 5B) and correspond to the 14-3-3/pTau complexes. Right, purified final preparations from triple (III) and dual (II) co-expression representing 14-3-3σΔC/pTau complex or pTau alone, respectively. On the last lane pTau^coex^ was loaded at twice as large amount (shown by multiple arrows to reflect its multisite phosphorylation). Note, that free 14-3-3 is absent in the sample III. For details, see also Materials and methods. The gel were stained by Coomassie brilliant blue.

Different 14-3-3 isoforms were found in deposits containing hyperphosphorylated Tau protein characteristic to tauopathies [58,59], suggesting that they can interact with phosphorylated Tau. Importantly, despite significant electrostatic attraction of Tau (alkaline pI) and 14-3-3 (acidic pI), these proteins only weakly interact under near physiological salt concentrations if Tau is not phosphorylated, whereas phosphorylation at specific sites triggers their tight binding even at higher salt concentrations [29,30]. Although the direct interaction of pTau with 14-3-3ζ isoform was investigated before [28–30,60], the data showing 14-3-3 isoform specificity in this interaction have not been presented so far. To address this question, we analyzed interaction of pTau^coex^ with the excess amounts of 14-3-3ε, γ, ζ, and σΔC isoforms using native gel-electrophoresis (Fig 6, left). In analogy to Fig 5A and 5B, the experiment clearly showed that all tested variants were able to form stable complexes with pTau^coex^, which justify the use of our co-expression system for 14-3-3/phosphotarget studies. Of note, under conditions used, pTau^coex^ entered the gel and showed multiple regularly shifted bands (Fig 6, right, lane “II”), suggesting decrease of its pI below that of the electrophoretic system (8.6) and being in agreement with its multisite phosphorylation detected by MS/MS (Table 2).

### Triple co-expression of Tau protein with PKA and 14-3-3

**Fig 7:**
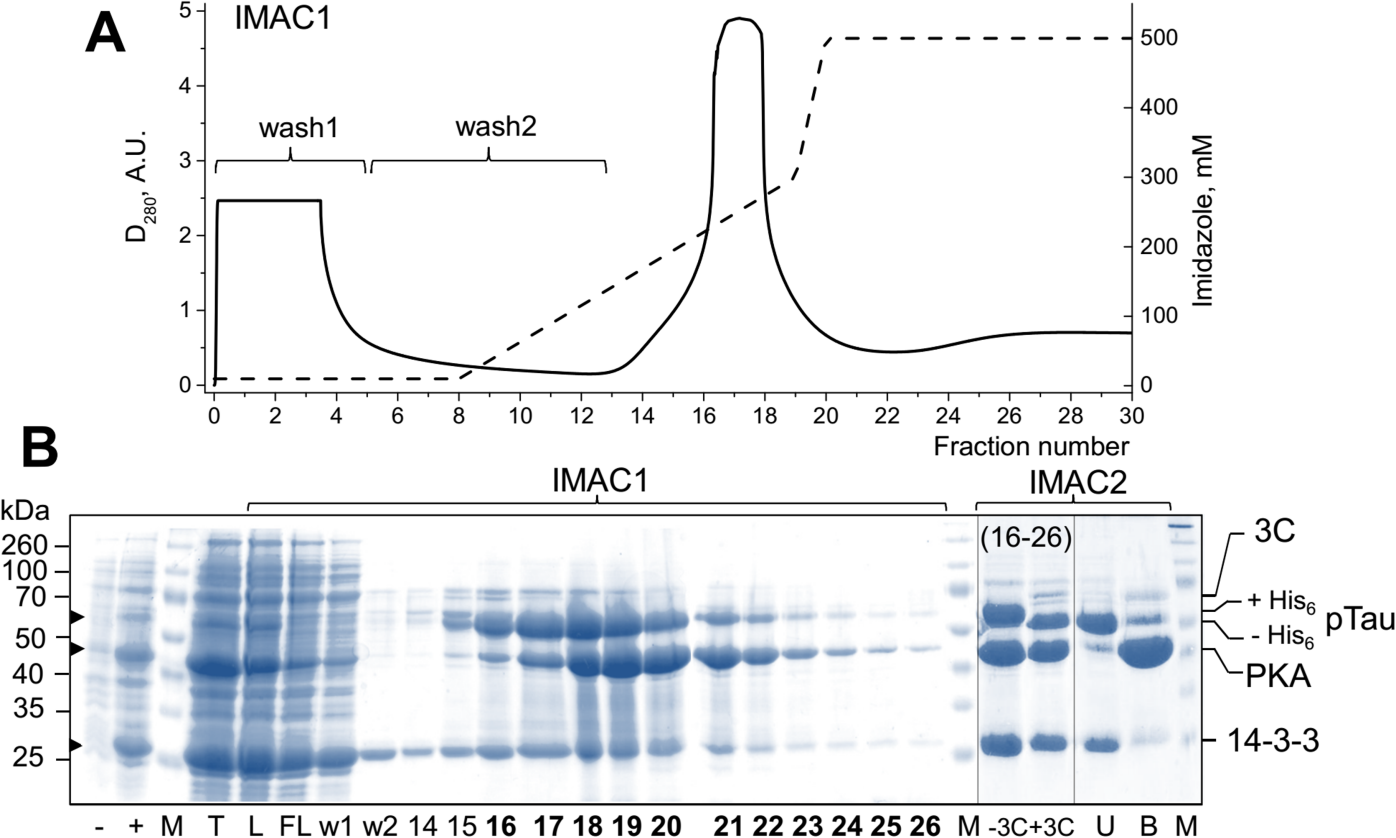
Co-purification of pTau and 14-3-3 using subtractive IMAC. **A.** IMAC1 profile obtained using a 10–500 mM imidazole gradient (dashed line) followed by absorbance at 280 nm (solid line) (**A**) with electrophoretic analysis of the fractions obtained (**B**, left). Arrows to the left indicate positions of 14-3-3 (26 kDa), PKA (45 kDa), pTau (52 kDa). Uninduced (-) and induced (+) sample; *IMAC1*: loaded sample (L), flowthrough (FL), wash 1 (fractions 1–5; w1) and wash 2 (fractions 6–13; w2), and fractions during elution (numbers) are indicated. Pooled fractions 16–26 (bold font) containing 14-3-3, PKA, and pTau (-3C) were dialyzed in the presence of 3C protease (+3C) to cleave off the His_6_-tag on pTau (the shift is indicated by positions of pTau with or without the His_6_-tag) and then subjected to *IMAC2*, with unbound (U; 20 mM imidazole) and bound (B; 500 mM imidazole) fractions collected. M, mass standards (in kDa).

Having established that pTau^coex^ tightly interacts with purified 14-3-3 proteins, thus recapitulating properties of the *in vitro* phosphorylated protein, we switched to the triple co-expression system. The competent BL21(DE3) cells expressing His_6_-tagged PKA and His_6_-3C-Tau were transformed with CDF-14-3-3σΔC plasmid to get simultaneous expression of Tau, PKA, and 14-3-3 (see Fig 1, right). After optimization of the growth and induction conditions, we obtained expression of all three components of the system, although in all cases His-tagged Tau was least abundant component (Fig 7). Co-expression of 14-3-3 did not affect phosphorylation pattern of Tau, as seen from mass-spectrometry identification of the same phosphopeptides as in the case of the dual co-expression of Tau and PKA (Table 2).

Similar to dual co-expression, PKA and Tau were bound to the Ni-Sepharose by its His-tags, and Tau was bound completely, as judged from its absence in the “flowthrough” fraction (Fig 7B). Expectedly, 14-3-3, which has no His-tag was unable to bind to the resin on its own (data not shown), but was co-purified with pTau throughout the IMAC1 profile despite the exhaustive washing step (see fractions “w1” and “w2”). This strongly suggested interaction between pTau and 14-3-3 upon co-expression and, apparently, it was preserved under conditions of lysis and IMAC1. Since a significant portion of 14-3-3 was found in the ‘flowthrough’ and ‘wash’ fractions, we concluded that Tau was expressed in substoichiometric amounts which ensured good conditions for its saturation with 14-3-3 lacking a His-tag. Under conditions used, we observed 14-3-3 in almost all fractions of pTau at near constant band intensities ratio, indicating the formation of stable complexes with a constant stoichiometry. We note that in general, parameters for a 14-3-3/phosphotarget co-purification on the metal-affinity chromatography should be optimized for each specific case, depending on a 14-3-3/partner binding affinity.

To cleave the His-tag of pTau protein, the eluate that contains proteins bound to the Ni-Sepharose (pTau, PKA, 14-3-3) was treated with the His-tagged 3C protease (Fig 1 and Fig 7B; “-3C”, “+3C”). After removal of imidazole by dialysis, the eluate was reloaded on the HisTrap column (IMAC2, see Fig 1 and Fig 7B; “IMAC2”). As expected, the 3C cleavage resulted in a shift of the Tau protein band toward lower masses (~1.6 kDa calculated mass difference) and in migration of both, pTau and 14-3-3, to the ‘flowthrough’ fraction. His_6_-tagged PKA and 3C, as well as contaminating proteins again bound to the resin (Fig 7B; “IMAC2”, lane “B”). The second IMAC step was found to be very efficient, yielding a pure mixture of pTau and 14-3-3 (Fig 7B; IMAC2, lane “U”), already in low imidazole buffer. Significantly, by performing its electrophoretic analysis under non-denaturing conditions, we observed the 14-3-3/pTau complex, very similar to that formed *in vitro* and no excess of 14-3-3 was detected (Fig 6, right, lane “III”). This sample containing purified 14-3-3/phospho-Tau complex formed upon triple bacterial co-expression of 14-3-3, its target, and phosphorylating kinase can be ultimately polished by preparative size-exclusion chromatography and then directly used for further structural characterization.

## Conclusions and perspectives

This study is devoted to the co-expression system for efficient and convenient production of phosphorylated Tau protein competent for 14-3-3 binding and, most importantly, for simultaneous expression of Tau, phosphorylating kinase (PKA), and 14-3-3 for biochemical and/or structural characterization. By applying this co-expression system to the well-established 14-3-3/pTau interaction [17,18,28–30], we could detect phosphorylation of already known and also novel potential 14-3-3-binding sites, whose phosphorylation has been reported, but physiological role as 14-3-3-binding sites has not been considered. Tau protein co-expressed with PKA displayed tight functional interaction with 14-3-3 isoforms of a different type. The system is in principle also applicable for other selected 14-3-3 targets, for example, for the less characterized Tau homolog, microtubule-associated protein 2c (MAP2c) [61], and has the following advantages: (i) a native-like crowded environment for target protein phosphorylation and 14-3-3 binding, (ii) a flexible and versatile system to obtain phosphotarget(s) or 14-3-3/phosphotarget complexes in one go with no need of separate purification of several components (target, kinase, 14-3-3) and tedious setting up conditions for *in vitro* phosphorylation and complex assembly, (iii) easy means to obtain 14-3-3/phosphotarget (stoichiometric) complexes to be directly used for biochemical and structural biology studies. The flexibility of the system is underlined by the fact that any its element (target, kinase, 14-3-3 type, availability of specific positions for phosphorylation) can be altered and further upgraded to improve performance. The system is currently tailored for utilization of only 14-3-3 isoforms naturally resistant to PKA phosphorylation-induced dimer dissociation, however, mutations of other 14-3-3 isoforms blocking site-specific phosphorylation should help in overcoming this limitation. The system described currently uses PKA to phosphorylate a 14-3-3 partner. While other protein kinases may also be used, it may be challenging to (co-)express them in a soluble, correctly folded form in bacteria. Nevertheless, it is possible to engineer protein partner’s phosphorylatable sites to facilitate their phosphorylation by PKA, provided that particular positions are known to be phosphorylated (by any kinase) and recognized by 14-3-3 members *in vivo*. Successful examples of such engineering to enforce PKA phosphorylation of a certain site can be found in literature [62]. As a limitation, the extent and identity of the sites phosphorylated by heterologous PKA *in bacteria* should be checked for each particular partner and, although somewhat difficult to control, may be steered by engineering (e.g., switching off) of undesired site(s). Still, this can be turned to an advantage of the possibility to separately analyze the role for particular sites in 14-3-3 binding. The introduced co-expression system seems promising for validation of newly identified (by other methods) 14-3-3 partners in both low and high-throughput format, provided that the protocol for co-purification of 14-3-3 complexes is suitably adjusted for selected partners.
